# VHA-guided resuscitation and post-24-hour survival in traumatic hemorrhage: a propensity- matched retrospective cohort study from China

**DOI:** 10.3389/fmed.2026.1865755

**Published:** 2026-07-16

**Authors:** Lincui Zhong, Qingwei Lin, Xiaomin Song, Qingbo Zeng, Longping He, Jingchun Song

**Affiliations:** 1 Intensive Care Unit, The 908th Hospital of Chinese PLA Logistic Support Force, Nanchang, China; 2 Nanchang Key Laboratory of Thrombosis and Hemostasis, Nanchang, China; 3 Intensive Care Unit, Nanchang Hongdou Hospital of Traditional Chinese Medicine, Nanchang, Jiangxi, China

**Keywords:** hemorrhage, hemostatic resuscitation, severe trauma, thromboelastography, transfusion

## Abstract

**Objective:**

Traumatic hemorrhage is the predominant preventable cause of mortality in severe trauma cases. Timely and efficacious hemostatic resuscitation is crucial for the management of severe traumatic hemorrhages. This study aimed to evaluate the association between implementation of viscoelastic hemostatic assay (VHA)/TEG-guided hemostatic resuscitation and clinical outcomes among transfused traumatic hemorrhage patients who survived at least 24 h after emergency department arrival in China.

**Methods:**

We conducted a retrospective, single-center before-after cohort study of trauma patients admitted to the emergency department of a tertiary trauma center from January 2012 to December 2021. Patients admitted from 2012 to 2016 were managed using a conventional coagulation assay (CCA)-guided strategy, whereas patients admitted from 2017 to 2021 were managed using a VHA/TEG-guided strategy. Because the analysis required complete 24-h coagulation and transfusion-response data, the primary cohort was defined as transfused traumatic hemorrhage patients who survived at least 24 h after emergency department arrival. Propensity score matching was used to reduce baseline imbalance. The primary outcome was post-24-h in-hospital mortality.

**Results:**

A total of 154 patients who survived at least 24 h were included after propensity score matching, with 77 patients in each group. Post-24-h in-hospital death occurred in 21 of 77 patients in the CCA-guided group and 9 of 77 patients in the VHA-guided group. Kaplan–Meier analysis showed improved post-24-h in-hospital survival in the VHA-guided group (log-rank *P* = 0.015). In multivariable Cox regression adjusting for age, ISS, APACHE II score, admission shock index, and baseline PT, VHA-guided resuscitation was associated with a lower hazard of post-24-h in-hospital death (adjusted HR 0.37, 95% CI 0.16–0.84, *P* = 0.018). The VHA-guided group exhibited reduced plasma utilization and greater fibrinogen-directed replacement compared with the CCA-guided group.

**Conclusion:**

Among transfused traumatic hemorrhage patients who survived at least 24 h, VHA-guided hemostatic resuscitation was associated with improved post-24-h in-hospital survival, reduced plasma exposure, greater fibrinogen-directed replacement, and improved PT correction. This study cannot determine whether VHA-guided resuscitation improves survival during the initial 24 h, when early hemorrhagic mortality is highest. Because of the retrospective before-after design, survivorship bias, and residual confounding, these findings should be interpreted as hypothesis-generating.

## 1 Introduction

Trauma stands as the predominant cause of mortality among individuals aged 10–49 years worldwide, accounting for an estimated 5 million deaths annually and contributing to 10% of the global disease burden (1, 2). Hemorrhage subsequent to traumatic injury is responsible for more than half of these fatalities, highlighting it as one of the most preventable causes of trauma-related mortality (3). Profuse bleeding can result in a significant depletion of coagulation factors and fibrinogen, induce secondary hyperfibrinolysis, cause extensive endothelial cell damage, and lead to endogenous heparinization, all of which can exacerbate hemorrhagic events and precipitate shock (4, 5). The time interval between injury and the control of hemorrhage is a critical determinant of patient outcomes (6). Consequently, hemorrhage resuscitation has become a central intervention in the management of trauma patients, although the strategies employed to guide these efforts exhibit considerable variation (7).

Early initiation of coagulation resuscitation is advised for patients with hemorrhage, with concurrent administration of red blood cells (RBCs), plasma, and platelets until laboratory coagulation tests are available (8, 9). Subsequently, coagulation management may be guided by conventional coagulation assays (CCA) or viscoelastic hemostatic assays (VHA) (10). CCA, including prothrombin time (PT), activated partial thromboplastin time (APTT), and Clauss fibrinogen levels, are based on a plasma-based coagulation model (11). In contrast, VHAs, such as thromboelastography (TEG), are performed in whole blood and provide dynamic information regarding clot initiation, clot strength, and fibrinolysis (12). Both approaches have been used to guide hemostatic therapy in traumatic hemorrhage (13–16). The iTACTIC trial did not show an overall mortality benefit of VHA-augmented protocols compared with conventional coagulation-test-guided management in major trauma hemorrhage (17), whereas other studies, including RETIC, suggested potential benefits of factor concentrate-based or VHA-guided approaches in selected settings (18). Evidence from Chinese trauma populations remains limited. Therefore, we conducted a single-center retrospective before-after study to evaluate whether implementation of VHA/TEG-guided hemostatic resuscitation was associated with improved outcomes among transfused traumatic hemorrhage patients in China.

## 2 Materials and methods

### 2.1 Study design and patient

This retrospective, single-center before-after cohort study included trauma patients admitted to the emergency department of our tertiary care facility in Nanchang, China, from January 2012 to December 2021. Eligibility criteria were: (1) injuries affecting at least two distinct body regions; (2) clinical manifestations indicative of hemorrhage; and (3) initial administration of 1 g tranexamic acid. Exclusion criteria were age below 18 years, congenital coagulopathy, chronic liver or renal disease, concurrent use of anticoagulants or antiplatelet agents, absence of transfusion requirements, death within 24 h after emergency department arrival, and incomplete medical records. Because the present analysis required complete 24-h coagulation and transfusion-response data, patients who died within the first 24 h were excluded from the primary analysis. Therefore, the study population should be interpreted as a 24-h landmark cohort of transfused traumatic hemorrhage patients who survived at least 24 h. The study was not designed to evaluate the effect of VHA-guided resuscitation on early hemorrhagic death within the first 24 h.

**FIGURE 1:**
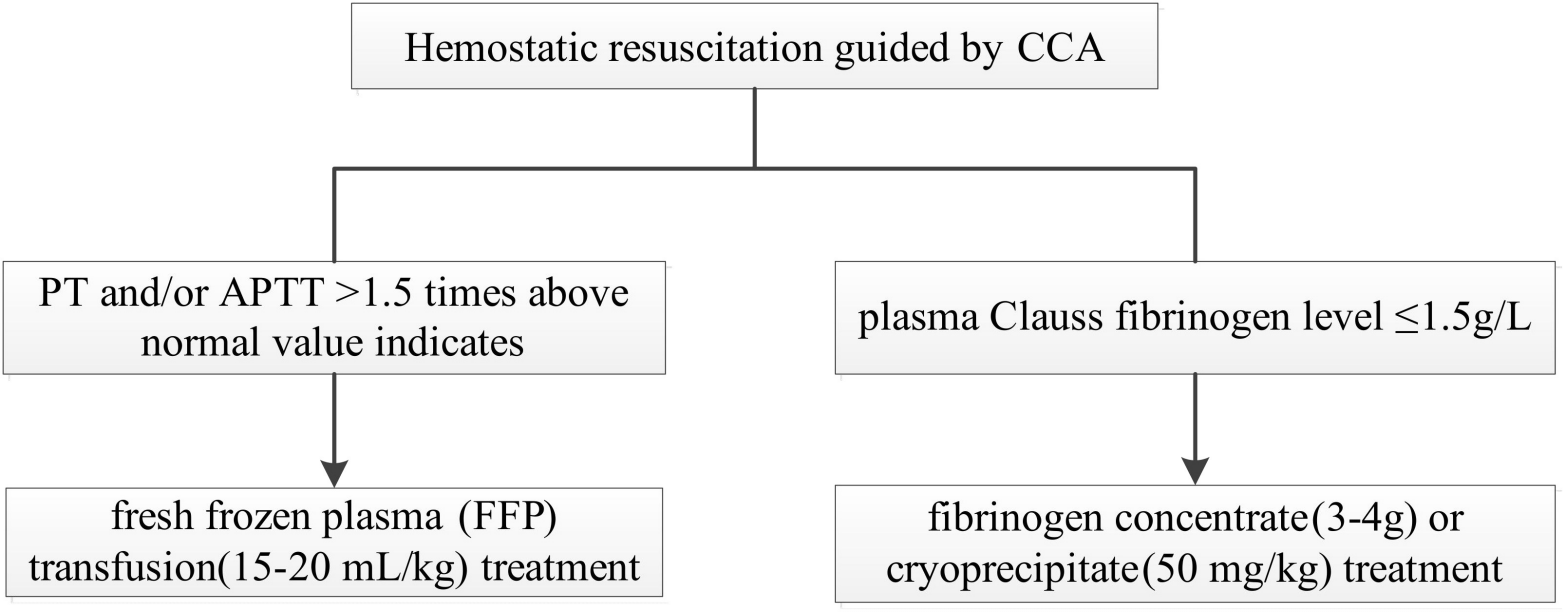
Flowchart of hemostatic resuscitation guided by CCA.

**FIGURE 2:**
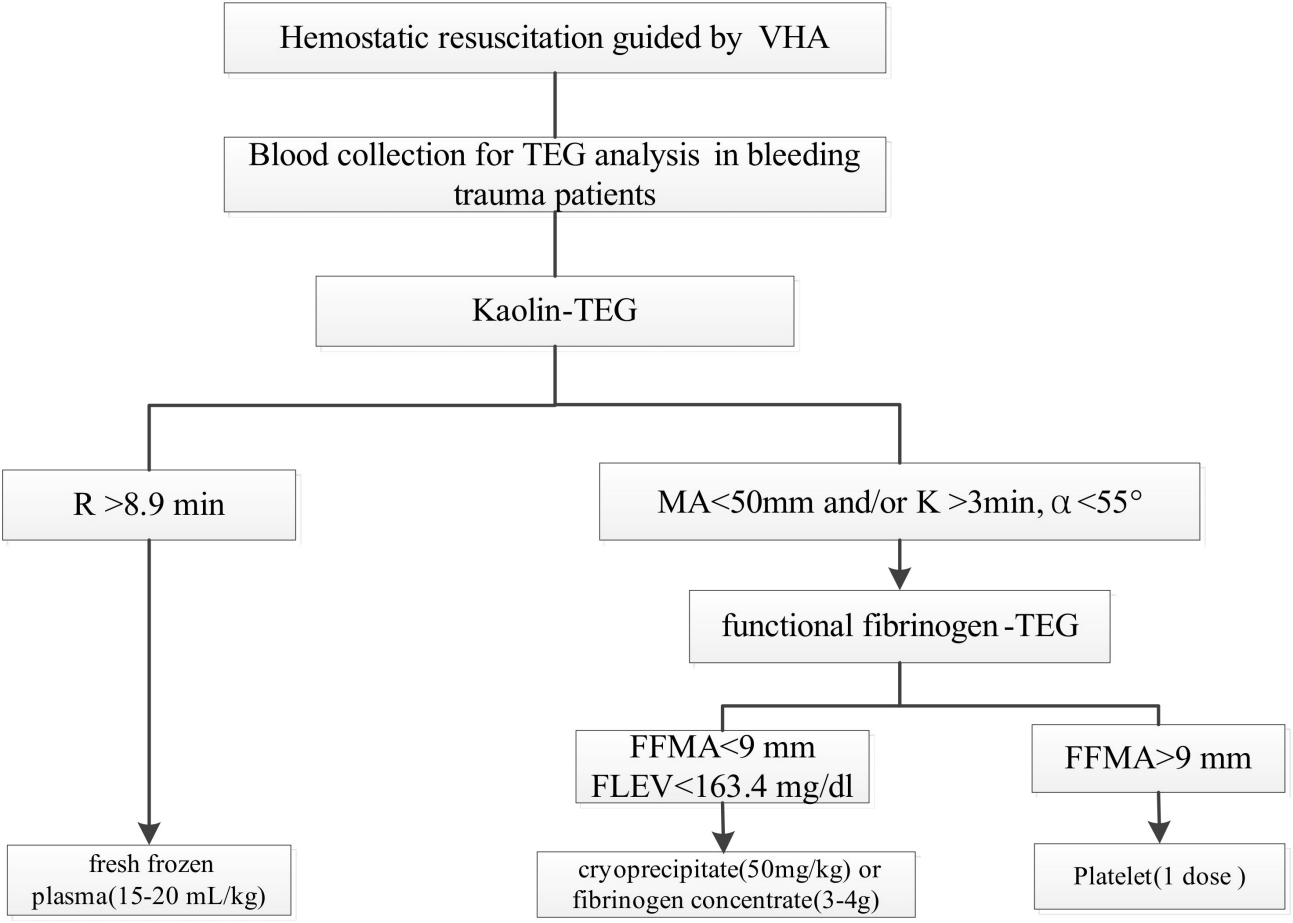
Flowchart of hemostatic resuscitation guided by TEG.

Patients admitted from January 2012 to December 2016 were classified into the CCA-guided group, whose hemostatic resuscitation was guided by conventional coagulation tests (Figure 1). In patients with significant hemorrhage, PT and/or APTT greater than 1.5 times the normal range triggered fresh frozen plasma administration, whereas a Clauss fibrinogen level < = 1.5 g/L guided fibrinogen concentrate or cryoprecipitate supplementation (19). Patients admitted from January 2017 to December 2021 were classified into the VHA-guided group, whose hemostatic resuscitation was informed by a TEG-guided algorithm (Figure 2). In this cohort, prolonged R time guided plasma administration, whereas K time, alpha angle, maximum amplitude, and functional fibrinogen were used to guide cryoprecipitate, platelet, and fibrinogen-directed therapy where appropriate. Both groups received early hemorrhage control, tranexamic acid administration, blood product support, warming, correction of acidosis, and calcium replacement when clinically indicated. Ethical approval for this study was granted by the Institutional Review Board of our hospital (approval number: 908yyLL028), with a waiver for informed consent.

### 2.2 Data collection

The following variables were derived from the medical records: age, sex, time interval from injury to emergency department arrival, mechanism of injury, mechanical ventilation, continuous renal replacement therapy, heart rate (HR), Shock Index (defined as HR divided by systolic blood pressure), Acute Physiology and Chronic Health Evaluation II (APACHE II) score (20), Injury Severity Score (ISS) (21), blood products transfused within the first 24 h, total duration of hospital stay, in-hospital mortality, white blood cell count, hemoglobin, hematocrit, platelet count, PT, APTT, fibrinogen, and creatinine levels at emergency department arrival and 24 h after admission.

### 2.3 Outcomes

The primary outcome was post-24-h in-hospital mortality among transfused traumatic hemorrhage patients who survived at least 24 h and had complete 24-h coagulation and transfusion-response data. Secondary outcomes included blood product utilization within the first 24 h, fibrinogen supplementation, changes in laboratory and coagulation parameters from admission to 24 h after admission, and hospital length of stay. Laboratory ratios were calculated as the 24-h value divided by the admission value. Because of the retrospective nature of the study, no formal prospective sample-size calculation was performed.

### 2.4 Statistical analysis

All statistical analyses were conducted utilizing R version 4.2.1 (R Core Team, Vienna, Austria) and GraphPad Prism version 8.0 (GraphPad Software Inc., La Jolla, CA, USA). Continuous variables were summarized as mean +/− standard deviation or median with interquartile range, as appropriate. Categorical variables were summarized as counts and percentages. Between-group comparisons were performed using Student’s *t*-test or the Mann–Whitney *U* test for continuous variables and the Chi-square test or Fisher’s exact test for categorical variables. A two-sided *P* value < 0.05 was considered statistically significant. To reduce baseline imbalance between groups, propensity scores were estimated using a multivariable logistic regression model with treatment group as the dependent variable. One-to-one nearest-neighbor matching without replacement was performed. Covariate balance before and after matching was assessed using standardized mean differences (SMDs), with an absolute SMD <0.1 generally considered acceptable. A Love plot and detailed SMD table were generated to show covariate balance. Kaplan-Meier curves were used to compare post-24-h in-hospital survival between groups, and differences were assessed using the log-rank test. Multivariable Cox proportional hazards regression was performed to evaluate the association between VHA-guided resuscitation and post-24-h in-hospital mortality after adjustment for residual confounders, including age, ISS, APACHE II score, admission shock index, and baseline PT. Results are reported as hazard ratios (HRs) with 95% confidence intervals (CIs).

## 3 Results

### 3.1 Demographic characteristics

**FIGURE 3:**
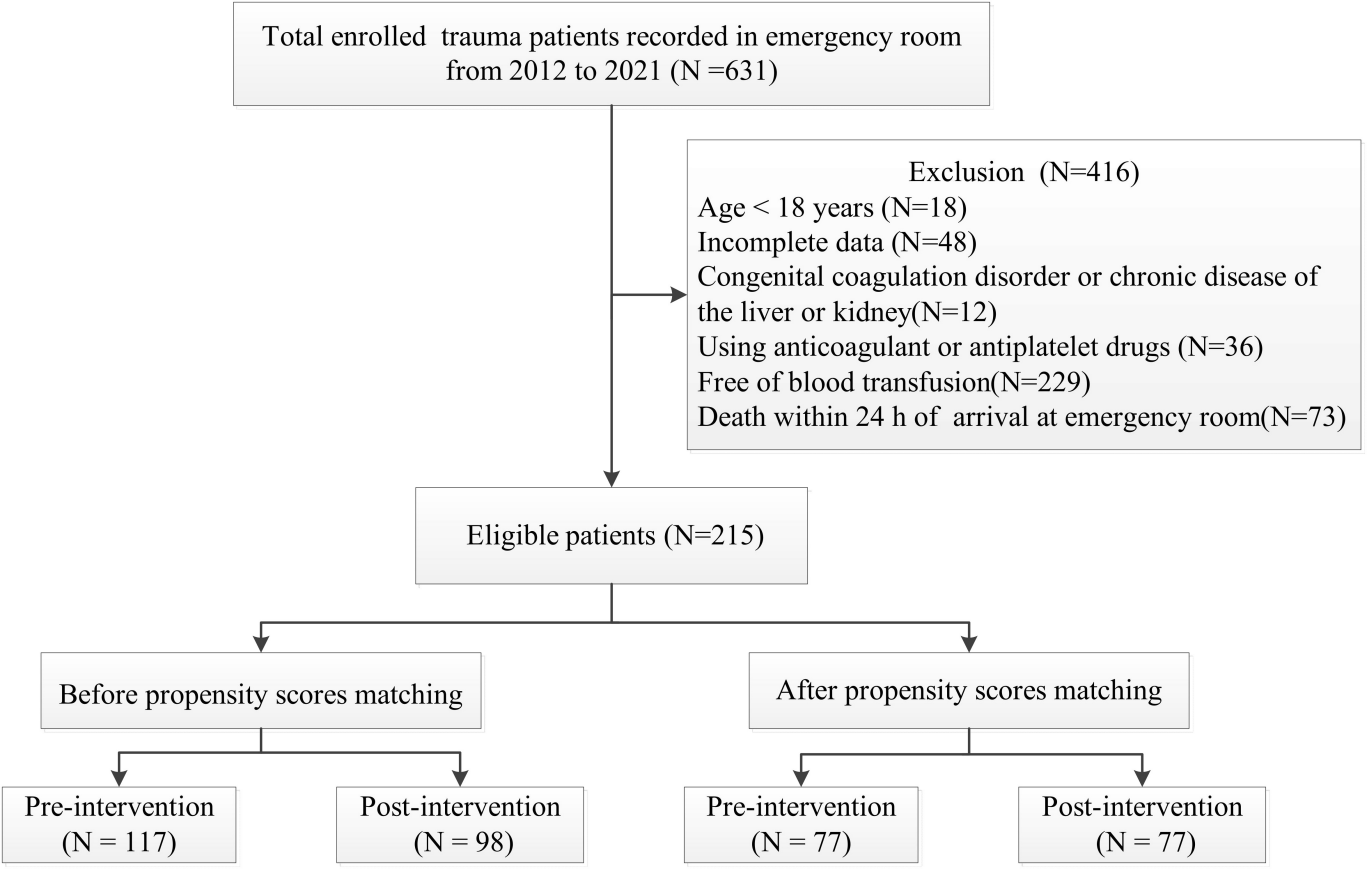
Flow diagram.

We excluded 18 patients who were under 18 years of age, 48 patients with incomplete data, 12 patients with congenital coagulation disorders or chronic liver or kidney disease, 36 patients with a history of anticoagulant or antiplatelet therapy, 73 patients who died within 24 h of arrival, and 229 patients who did not receive a blood transfusion. A total of 215 patients were included in the primary 24-h landmark analysis (Figure 3). Before propensity score matching, the CCA-guided and VHA-guided groups differed in several baseline characteristics, including age, ISS, shock index, and baseline PT. After 1:1 propensity score matching, 154 patients remained, with 77 patients in each group. Baseline characteristics and standardized mean differences before and after matching are shown in Table 1, and detailed balance diagnostics are provided in Supplementary Figure 1. After propensity score matching, most baseline covariates achieved acceptable balance (absolute SMD <0.1). However, residual imbalance persisted for baseline PT (SMD = 0.149; CCA-guided: 17.7 ± 11.0 s vs. VHA-guided: 19.4 ± 11.3 s), which was therefore included as a covariate in the subsequent multivariable Cox regression model.

**TABLE 1:** Demographics, injuries characteristics and initial therapies.

	Before propensity scores matching				After propensity scores matching			
Characteristics	CCA-guided group	VHA-guided group	*P* value	SMD	CCA-guided group	VHA-guided group	*P* value	SMD
	(*n* = 117)	(*n* = 98)			(*n* = 77)	(*n* = 77)		
Age (years)	47 ± 16	51 ± 15	0.036	0.288	51 ± 16	49 ± 14	0.416	0.131
Sex			0.789	0.037			1	0.000
Female	28 (24%)	25 (26%)			20 (26%)	20 (26%)		
Male	89 (76%)	73 (74%)			57 (74%)	57 (74%)		
Mechanism of injury			0.551	0.082			0.772	0.047
Sharp instrument injury	11 (9)	7 (7)			6 (8)	7 (9)		
Blunt force injury	106 (91)	91 (93)			71 (92)	70 (91)		
Time to admission (hours)	6 (4, 10)	8 (4, 14)	0.067	0.221	6 (3, 10)	8 (4, 14)	0.067	0.307
Heart rate (beats per minute)	97 (78, 116)	105 (80, 120)	0.190	0.166	99 (80, 113)	105 (80, 120)	0.155	0.220
Shock index	0.9 (0.7, 1.1)	1 (0.72, 1.2)	0.045	0.233	0.9 (0.7, 1.1)	1.0 (0.7, 1.2)	0.3	0.177
White blood cells( × 109/L)	12.8 (8.9, 16.8)	11.5 (7.9, 15.6)	0.183	0.209	12.8 (9.7, 16.9)	11.3 (7.9, 15.2)	0.121	0.271
Hemoglobin(g/L)	93.0 ± 25.9	86.2 ± 25.2	0.053	0.267	94.8 ± 25.6	88.2 ± 26.9	0.126	0.248
Hematocrit (%)	27.1 (22.0, 32.6)	25.6 (20.7, 31.4)	0.223	0.201	27.5 (22.4, 35.5)	25.7 (20.7, 33.7)	0.319	0.210
platelet count (×10^9^/L)	92.0 (61.5, 129.0)	94.0 (60.0, 129.3)	0.747	0.091	92.0 (62.0, 129.5)	101.0 (56.5, 140.5)	0.937	0.020
PT (s)	14.8 (13.8, 17.8)	16.9 (14.2, 21.9)	0.011	0.292	14.6 (13.9, 17.0)	16.7 (14.1, 22.1)	0.023	0.149
APTT (s)	38.5 (32.7, 49.0)	40.0 (31.6, 49.7)	0.696	0.137	38.3 (33.2, 49.4)	38.2 (30.5, 51.3)	0.759	0.015
Fibrinogen (g/L)	1.47 (1.15, 1.81)	1.42 (0.95, 2.21)	0.719	0.101	1.47 (1.17, 1.77)	1.43(0.95,2.32)	0.766	0.070
Creatinine (μg/mL)	88.1 (70.2, 103.9)	85.8 (63.0, 122.9)	0.908	0.119	87.7 (68.8, 102.2)	83.6 (62.4, 121.1)	0.919	0.144
APACHE II (score)	22 (18, 27)	22 (18, 26)	0.977	0.050	22.84 ± 8.37	21.65 ± 6.57	0.326	0.159
ISS (score)	29 (22, 34)	32 (24, 43)	0.017	0.394	30 (27, 34)	29 (24, 41)	0.676	0.122
Mechanical ventilation	90 (77%)	86 (88%)	0.04	0.284	58 (75%)	67 (87%)	0.064	0.299
Continuous renal replacement therapy	8 (7%)	15 (15%)	0.045	0.270	3 (4%)	11 (14%)	0.046	0.361

### 3.2 Post-24-h in-hospital survival: CCA-guided versus VHA-guided groups

**FIGURE 4:**
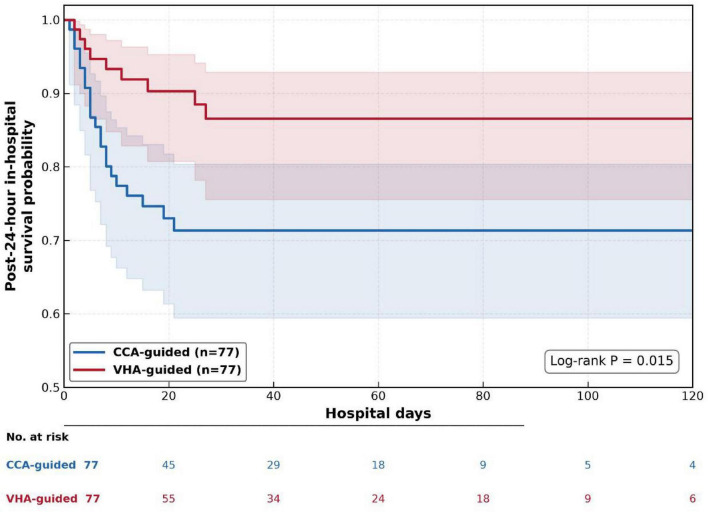
Kaplan–Meier analysis of post-24-h in-hospital survival after propensity score matching. The matched cohort included 77 patients in each group. Post-24-h in-hospital death occurred in 21/77 patients in the CCA-guided group and 9/77 patients in the VHA-guided group. Log-rank *P* = 0.015.

In the matched cohort, post-24-h in-hospital death occurred in 21 of 77 patients in the CCA-guided group and 9 of 77 patients in the VHA-guided group. Kaplan-Meier analysis showed higher post-24-h in-hospital survival in the VHA-guided group than in the CCA-guided group (log-rank *P* = 0.015; Figure 4). In multivariable Cox regression adjusting for age, ISS, APACHE II score, admission shock index, and baseline PT, VHA-guided resuscitation remained associated with a lower hazard of post-24-h in-hospital death (adjusted HR 0.37, 95% CI 0.16–0.84, *P* = 0.018; Table 2).

**TABLE 2:** Blood transfusion within the first 24 h after admission at emergency room.

Characteristics	CCA-guided group	VHA-guided group	*P*
Fresh frozen plasma			
Treated patients	77 (100%)	64 (83.1%)	<0.001
Dose (ml)	600 (350, 800)	400 (200, 860)	0.091
Red blood cell concentrate			
Treated patients	67 (87.0%)	62 (80.5%)	0.275
Dose (IU)	4 (2, 8)	4 (1, 7.5)	0.245
Platelet			
Treated patients	48 (62.3%)	45 (58.4%)	0.621
Dose (ml)	200 (0, 250)	200 (0, 250)	0.906
Cryoprecipitate			
Treated patients	44 (57.1%)	55 (71.4%)	0.064
Dose (IU)	10 (0, 15)	10 (0, 20)	0.042
Fibrinogen concentrate			
Treated patients	0	20 (26%)	<0.001
Dose (g)	0	0 (0, 0.75)	<0.001

### 3.3 Blood product utilization within the first 24 h after admission

Table 2 displays the data regarding the quantity of blood transfusions carried out within the initial 24 h post-emergency room admission. Across both cohorts, patients uniformly received 4 IU of red blood cell concentrate, with no significant variation in transfusion rates (87.0% vs. 80.5%, *P* = 0.275). For platelet transfusions, the median volume administered was 200 ml in both groups, with no significant difference in transfusion rates (62.3% vs. 58.4%, *P* = 0.621). It is noteworthy that the VHA-guided group had a significantly lower frequency of FFP transfusions compared to the CCA-guided group (83.1% vs. 100%, P < 0.001), despite no significant difference in the volume of FFP transfused between the groups [400 (200, 860) ml vs. 600 (350, 800) ml, *P* = 0.091]. Moreover, the VHA-guided group showed a higher volume of cryoprecipitate transfusions compared to the CCA-guided group (*P* = 0.042), although there was no significant difference in the frequency of cryoprecipitate transfusions between the groups (*P* = 0.064). Additionally, the VHA-guided group more frequently utilized fibrinogen concentrate (0% vs. 26%, *P* < 0.001).

### 3.4 Changes in laboratory parameters from admission to 24 h after admission

Laboratory ratios were calculated by dividing the 24-h value by the admission value. As shown in Table 3, the PT ratio was lower in the VHA-guided group than in the CCA-guided group [0.86 (0.70, 0.98) vs. 0.95 (0.84, 1.01), *P* = 0.016], suggesting greater improvement in PT over the first 24 h. No significant between-group differences were observed for the ratios of WBC, hemoglobin, hematocrit, platelet count, APTT, fibrinogen, or creatinine.

**TABLE 3:** Coagulation parameters ratio before and after 24 h of admission between CCA-guided group and VHA-guided group.

Characteristics	CCA-guided group	VHA-guided group	*P*
WBC ratio	0.98 (0.74, 1.29)	0.99 (0.71, 1.28)	0.896
Hemoglobin ratio	0.94 (0.80, 1.10)	0.90 (0.80, 1.06)	0.454
Hematocrit ratio	0.95 (0.79, 1.07)	0.89 (0.79, 1.05)	0.375
Platelet count ratio	0.79 (0.56, 1.06)	0.88 (0.63, 1.21)	0.120
PT ratio	0.95 (0.84, 1.01)	0.86 (0.70, 0.98)	0.016
APTT ratio	0.94 (0.72, 1.10)	0.89 (0.71, 1.03)	0.603
Fibrinogen ratio	1.72 (1.35, 2.15)	1.95 (1.35, 2.66)	0.193
Creatinine ratio	1.02 (0.84, 1.19)	0.95 (0.76, 1.11)	0.137

## 4 Discussion

In this retrospective, single-center before-after cohort study of transfused traumatic hemorrhage patients who survived at least 24 h, implementation of VHA/TEG-guided hemostatic resuscitation was associated with higher post-24-h in-hospital survival, reduced plasma exposure, greater fibrinogen-directed replacement, and improved PT correction compared with CCA-guided resuscitation. Both CCA and VHA are endorsed for directing coagulation resuscitation in individuals experiencing massive hemorrhage or traumatic coagulopathy (22). Within the CCA framework, plasma fibrinogen levels, PT, and APTT are the parameters most commonly employed. PT measures the time required for blood to clot, primarily evaluating the extrinsic pathway of the coagulation cascade. APTT assesses the intrinsic and common pathways of the coagulation cascade, evaluating factors such as VIII, IX, XI, and XII, as well as fibrinogen. Clauss fibrinogen levels provide a clot-based functional assay of fibrinogen. In the context of severe traumatic hemorrhage, fibrinogen levels are observed to plummet rapidly, while PT and APTT are elongated as a result of coagulation factor depletion and extensive thrombus formation (23). Clinicians often administer FFP or cryoprecipitate to trauma patients based on these aberrant coagulation indices (24). However, PT and APTT can frequently manifest pseudo-prolongation in the setting of significant hypofibrinogenemia, which may precipitate excessive plasma utilization and delayed correction of hypofibrinogenemia (25). TEG, due to its distinct detection mechanisms, can effectively differentiate between coagulation factor impairment, denoted by an extended TEG-R time, and hypofibrinogenemia, which is indicated by an elongated TEG-K time or a reduced α angle (26). Therefore, a TEG-guided protocol can mitigate the overutilization of plasma and advocate for the timely administration of fibrinogen supplementation, potentially contributing to the enhanced hospital survival rates associated with VHA-guided resuscitation protocols.

Fibrinogen, a pivotal protein in the terminal phase of the coagulation cascade, is rapidly consumed during massive hemorrhage (27). The rapid replenishment of fibrinogen levels can be achieved through the administration of cryoprecipitate or fibrinogen concentrates (28). However, the current body of evidence does not robustly endorse the hypothesis that the use of cryoprecipitate or fibrinogen concentrate therapy enhances hospital survival rates among patients with traumatic massive hemorrhage (29). Nonetheless, fibrinogen concentrates are gaining popularity due to their ease of administration, absence of the need for blood type compatibility, and effective viral inactivation (30). Conversely, cryoprecipitates are posited to possess superior hemostatic properties, attributed to their higher concentration of coagulation factor XIII (31). Consequently, the timely administration of either cryoprecipitates or fibrinogen concentrates is essential in determining the clinical benefits for patients suffering from traumatic hemorrhage.

Prolonged PT following trauma indicates the activation of the extrinsic coagulation pathway in damaged tissues, which has been identified as an independent predictor of mortality in trauma cases (32). Our findings suggest that in the Viscoelastic Hemostatic Assay (VHA)-guided group, compared to the Conventional Coagulation Assay (CCA)-guided group, a decrease in the PT ratio within 24 h of admission indicates that VHA-targeted therapy may contribute to a more rapid improvement in coagulopathy. The underlying reason is that VHA exhibits high specificity in identifying hypofibrinogenemia, guiding trauma patients to receive fibrinogen concentrate treatment earlier, and timely correcting hypofibrinogenemia. This adjustment was associated with improved post-24-h prognosis among patients with traumatic hemorrhage and was also associated with reduced utilization of Fresh Frozen Plasma (FFP).

This study has several limitations. First, the retrospective before-after design compared two sequential historical periods; therefore, the observed association may reflect secular changes in trauma care and hemostatic product availability over the decade rather than the isolated effect of the VHA-guided strategy. Second, patients who died within 24 h were excluded owing to missing coagulation and transfusion data, introducing survivorship bias and limiting inference regarding early hemorrhagic mortality. Third, despite propensity score matching and multivariable adjustment, residual confounding cannot be excluded, as detailed injury characteristics and cause-specific mortality were not consistently recorded. Fourth, the introduction of fibrinogen concentrate coincided with VHA implementation, meaning that the study evaluates a bundled change in practice rather than the independent effect of VHA-guided resuscitation. Fifth, the single-center design and limited matched sample size (77 patients per group) restrict generalizability, and residual imbalance in baseline PT after matching (SMD = 0.149) may have contributed to the observed association. Prospective multicenter studies are required to confirm these hypothesis-generating findings.

## 5 Conclusions

In this single-center retrospective cohort study of trauma patients who survived at least 24 h, VHA-guided resuscitation was associated with improved survival, decreased plasma requirements, increased fibrinogen supplementation, and better PT correction. Importantly, given that fibrinogen concentrate was introduced concurrently with the VHA-guided protocol, the observed association represents a bundled practice change rather than the isolated effect of VHA implementation. These hypothesis-generating findings merit further validation in prospective multicenter studies.

## Data Availability

The raw data supporting the conclusions of this article will be made available by the authors, without undue reservation.

## References

[1] GBD 2019 Diseases and Injuries Collaborators. Global burden of 369 diseases and injuries in 204 countries and territories, 1990-2019: a systematic analysis for the global burden of disease study 2019. *Lancet.* (2020) 396:1204–22. 10.1016/S0140-6736(20)30925-9 33069326 PMC7567026

[2] GBD 2016 Causes of Death Collaborators. Global, regional, and national age-sex specific mortality for 264 causes of death, 1980-2016: a systematic analysis for the global burden of disease study 2016. *Lancet.* (2017) 390:1151–210. 10.1016/S0140-6736(17)32152-9 28919116 PMC5605883

[3] Brohi K Gruen RL Holcomb JB. Why are bleeding trauma patients still dying? *Intensive Care Med.* (2019) 45:709–11. 10.1007/s00134-019-05560-x 30741331

[4] James A Abback PS Pasquier P Ausset S Duranteau J Hoffmann C et al. The conundrum of the definition of haemorrhagic shock: a pragmatic exploration based on a scoping review, experts’ survey and a cohort analysis. *Eur J Trauma Emerg Surg.* (2022) 48:4639–49. 10.1007/s00068-022-01998-9 35732811 PMC9712310

[5] Ho VK Wong J Martinez A Winearls J. Trauma-induced coagulopathy: mechanisms and clinical management. *Ann Acad Med Singap.* (2022) 51:40–8. 10.47102/annals-acadmedsg.2020381 35091729

[6] Vulliamy P Thaventhiran AJ Davenport RA. What’s new for trauma haemorrhage management? *Br J Hosp Med.* (2019) 80:268–73. 10.12968/hmed.2019.80.5.268 31059346

[7] Gonzalez E Moore EE Moore HB Chapman MP Chin TL Ghasabyan A et al. Goal-directed hemostatic resuscitation of trauma-induced coagulopathy: a pragmatic randomized clinical trial comparing a viscoelastic assay to conventional coagulation assays. *Ann Surg.* (2016) 263:1051–9. 10.1097/SLA.0000000000001608 26720428 PMC5432433

[8] Holcomb JB Jenkins D Rhee P Johannigman J Mahoney P Mehta S et al. Damage control resuscitation: directly addressing the early coagulopathy of trauma. *J Trauma.* (2007) 62:307–10. 10.1097/TA.0b013e3180324124 17297317

[9] Henriksen HH Rahbar E Baer LA Holcomb JB Cotton BA Steinmetz J et al. Pre-hospital transfusion of plasma in hemorrhaging trauma patients independently improves hemostatic competence and acidosis. *Scand J Trauma Resusc Emerg Med.* (2016) 24:145. 10.1186/s13049-016-0327-z 27938373 PMC5148857

[10] Shammassian BH Ronald A Smith A Sajatovic M Mangat HS Kelly ML. Viscoelastic hemostatic assays and outcomes in traumatic brain injury: a systematic literature review. *World Neurosurg.* (2022) 159:221.e–36.e. 10.1016/j.wneu.2021.10.180 34844010

[11] Shah A McKechnie S Stanworth S. Use of plasma for acquired coagulation factor deficiencies in critical care. *Semin Thromb Hemost.* (2016) 42:95–101. 10.1055/s-0035-1564830 26716502

[12] Meizoso JP Barrett CD Moore EE Moore HB. Advances in the management of coagulopathy in trauma: the role of viscoelastic hemostatic assays across all phases of trauma care. *Semin Thromb Hemost.* (2022) 48:796–8 07. 10.1055/s-0042-1756305 36113505

[13] Nascimento B Callum J Tien H Rubenfeld G Pinto R Lin Y et al. Effect of a fixed-ratio (1:1:1) transfusion protocol versus laboratory-results-guided transfusion in patients with severe trauma: a randomised feasibility trial. *CMAJ.* (2013) 185:E583–9. 10.1503/cmaj.121986 23857856 PMC3761040

[14] Hilbert-Carius P Hofmann G Stuttmann R. Haemoglobin-oriented and coagulation factor-based algorithm: effect on transfusion needs and standardized mortality rate in massively transfused trauma patients. *Anaesthesist.* (2015) 64:828–38. 10.1007/s00101-015-0093-8 26453580

[15] Schöchl H Maegele M Voelckel W. Fixed ratio versus goal-directed therapy in trauma. *Curr Opin Anaesthesiol.* (2016) 29:234–44. 10.1097/ACO.0000000000000278 26595548

[16] Bainbridge FJ Sinha R Tocchetti R Clarke C Martin D Foo N et al. Introduction of point-of-care ROTEM testing in the emergency department of an Australian level 1 trauma centre and its effect on blood product use. *Emerg Med Australas.* (2021) 33:893–9. 10.1111/1742-6723.13767 33733606

[17] Baksaas-Aasen K Gall LS Stensballe J Juffermans NP Curry N Maegele M et al. Viscoelastic haemostatic assay augmented protocols for major trauma haemorrhage (ITACTIC): a randomized, controlled trial. *Intensive Care Med.* (2021) 47:49–59. 10.1007/s00134-020-06266-1 33048195 PMC7550843

[18] Innerhofer P Fries D Mittermayr M Innerhofer N von Langen D Hell T et al. Reversal of trauma-induced coagulopathy using first-line coagulation factor concentrates or fresh frozen plasma (RETIC): a single-centre, parallel-group, open-label, randomised trial. *Lancet Haematol.* (2017) 4:e258–71. 10.1016/S2352-3026(17)30077-7 28457980

[19] Spahn DR Bouillon B Cern Duranteau JV Maegele M Rossaint R Komadina R et al. The European guideline on management of major bleeding and coagulopathy following trauma: fifth edition. *Crit Care.* (2019) 23:98. 10.1186/s13054-019-2347-3 30917843 PMC6436241

[20] Le Gall J-R Loirat P Alpérovitch A. APACHE II–a severity of disease classification system. *Crit Care Med.* (1986) 14:754–5. 10.1097/00003246-198608000-00027 3087704

[21] Dai G Lu X Xu F Xu D Li P Chen X et al. Early mortality risk in acute trauma patients: predictive value of injury severity score. *J Clin Med.* (2022) 11:7219. 10.3390/jcm11237219 36498793 PMC9735436

[22] Rossaint R Afshari A Bouillon B Cerny V Cimpoesu D Curry N et al. The European guideline on management of major bleeding and coagulopathy following trauma: sixth edition. *Crit Care.* (2023) 27:80. 10.1186/s13054-023-04327-7 36859355 PMC9977110

[23] Kleinveld DJB Hamada SR Sandroni C. Trauma-induced coagulopathy. *Intensive Care Med.* (2022) 48:1642–5. 10.1007/s00134-022-06834-7 35925321

[24] Dilday J Lewis MR. Transfusion management in the trauma patient. *Curr Opin Crit Care* (2022) 28:725–31. 10.1097/MCC.0000000000000992 36226706

[25] Lawson MA Holle LA Dow NE Hennig G de Laat B Moore HB et al. Plasma-based assays distinguish hyperfibrinolysis and shutdown subgroups in trauma-induced coagulopathy. *J Trauma Acute Care Surg.* (2022) 93:579–87. 10.1097/TA.0000000000003723 35687811 PMC9613511

[26] Selby R. “TEG talk”: expanding clinical roles for thromboelastography and rotational thromboelastometry. *Hematology Am Soc Hematol Educ Program.* (2020) 2020:67–75. 10.1182/hematology.2020000090 33275705 PMC7727516

[27] Meizoso JP Moore EE Pieracci FM Saberi RA Ghasabyan A Chandler J et al. Role of fibrinogen in trauma-induced coagulopathy. *J Am Coll Surg.* (2022) 234:465–73. 10.1097/XCS.0000000000000078 35290265

[28] Peng HT Nascimento B Rhind SG da Luz L Beckett A. Evaluation of trauma-induced coagulopathy in the fibrinogen in the initial resuscitation of severe trauma trial. *Transfusion.* (2021) 61:S49–57. 10.1111/trf.16488 34269460

[29] Obaid O Anand T Nelson A Reina R Ditillo M Stewart C et al. Fibrinogen supplementation for the trauma patient: Should you choose fibrinogen concentrate over cryoprecipitate? *J Trauma Acute Care Surg.* (2022) 93:453–60. 10.1097/TA.0000000000003728 35838235

[30] Itagaki Y Hayakawa M Takahashi Y Hirano S Yamakawa K. Emergency administration of fibrinogen concentrate for haemorrhage: systematic review and meta-analysis. *World J Emerg Surg.* (2023) 18:27. 10.1186/s13017-023-00497-5 36998084 PMC10061696

[31] Ho D Chan E Campbell D Wake E Walters K Bulmer AC et al. Targeted cryoprecipitate transfusion in severe traumatic haemorrhage. *Injury.* (2020) 51:1949–55. 10.1016/j.injury.2020.05.044 32553426

[32] Hochart A Momal R Garrigue-Huet D Drumez E Susen S Bijok B. Prothrombin time ratio can predict mortality in severe pediatric trauma: study in a french trauma center level 1. *Am J Emerg Med.* (2020) 38:2041–4. 10.1016/j.ajem.2020.06.075 33142171

