# Supplementary material for: VHA-guided resuscitation and post-24-hour survival in traumatic hemorrhage: a propensity- matched retrospective cohort study from China
Source: Front Med (Lausanne). 2026 Jul 16;13:1865755. doi: 10.3389/fmed.2026.1865755 (PMC13422429; doi:10.3389/fmed.2026.1865755)
Supplement: Supplementary file 1 [file Data_Sheet_1.pdf]

**Supplementary Figure S1. Standardized mean differences before and after propensity score matching**

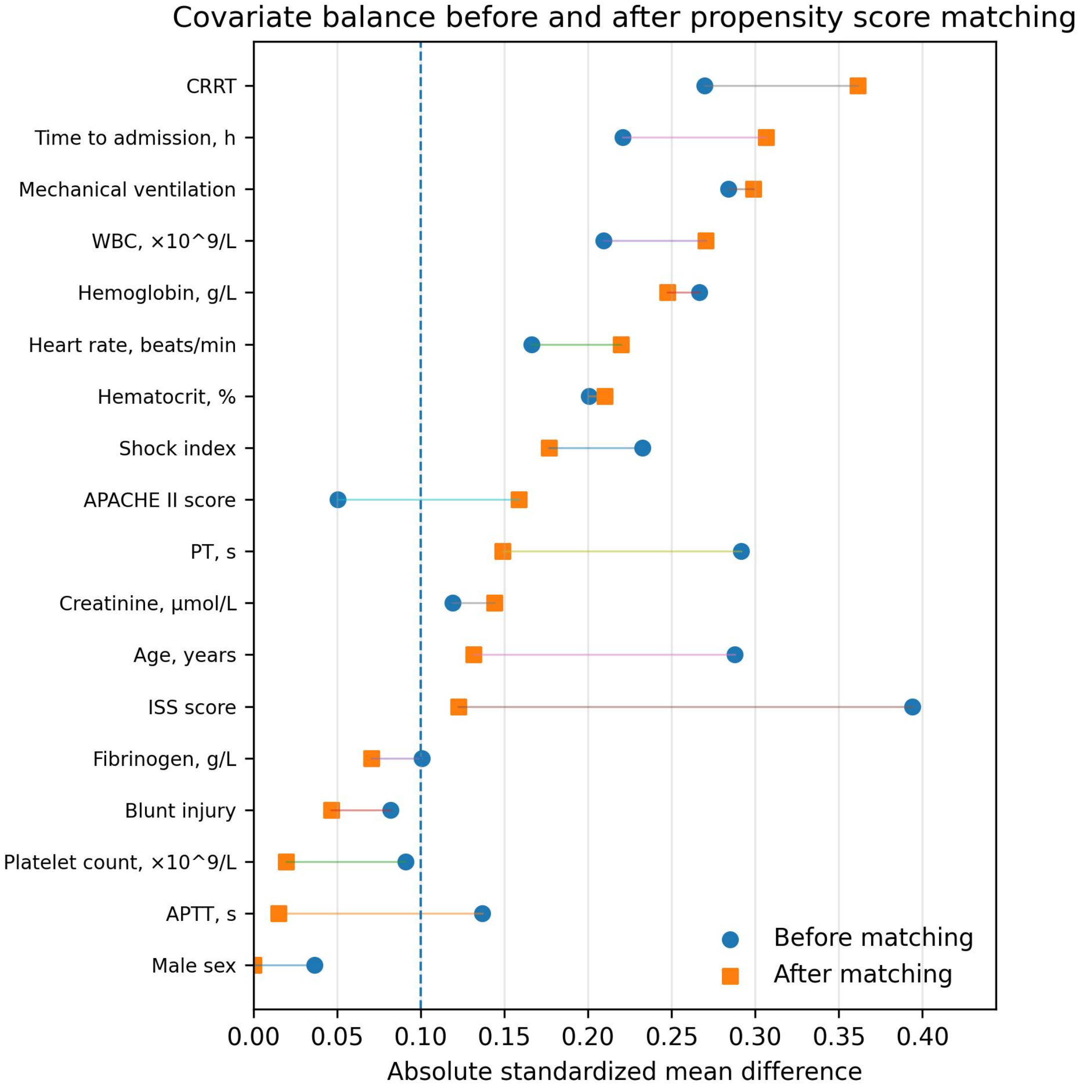


The dashed reference line indicates an absolute standardized mean difference of 0.10. Several variables remained partially imbalanced after matching; therefore, residual confounders were further adjusted in Cox regression.
